# Modeling and experimental investigation of the effect of carbon source on the performance of tubular microbial fuel cell

**DOI:** 10.1038/s41598-023-38215-5

**Published:** 2023-07-08

**Authors:** Masoud Karamzadeh, Milad Kadivarian, Peyman Mahmoodi, Seyedeh Sajedeh Asefi, Amirhossein Taghipour

**Affiliations:** 1 Department of Chemical Engineering, Iran University of Science and Technology, Tehran, Iran; 2 Department of Chemical Engineering, Isfahan University of Technology, Isfahan, 84156-83111 Iran; 3 Department of Chemical Engineering, Babol Noshirvani University of Technology, Babol, Iran

**Keywords:** Environmental biotechnology, Environmental chemistry, Environmental impact

## Abstract

Microbial fuel cells (MFCs) serve two main purposes: clean energy production and wastewater treatment. This study examines the impact of different carbon sources on MFC performance and develops a mathematical model to replicate the polarization curve. The biological reactor employed three types of carbon sources: glucose as a simple feed, microcrystalline cellulose (MCC), and a slurry of the organic component of municipal solid waste (SOMSW) as complex feeds. The MFCs were operated in both open and closed circuit modes. The maximum open circuit voltages achieved were 695 mV for glucose, 550 mV for MCC, and 520 mV for SOMSW as substrates. The influence of the substrate in closed circuit mode was also investigated, resulting in maximum power densities of 172 mW/m^2^, 55.5 mW/m^2^, and 47.9 mW/m^2^ for glucose, MCC, and SOMSW as substrates, respectively. In the second section, a mathematical model was developed to depict the polarization curve while considering voltage losses, namely activation, ohmic, and concentration loss, with an average relative error (ARE) of less than 10%. The mathematical models demonstrated that the activation loss of voltage increased with the complexity of the substrate and reached its peak value when SOMSW was used as the substrate.

## Introduction

In recent decades, the global demand for energy has witnessed a substantial surge, primarily driven by population growth and industrial advancements. Presently, the majority of energy needs are met by relying heavily on finite fossil fuel resources such as gas, oil, and coal. As energy consumption continues to escalate, there has been a growing competition among scientists to discover a renewable, environmentally friendly, and dependable energy alternative. It is imperative to address these concerns as fossil fuel sources are not only finite but also environmentally unsustainable. In addition to energy-related challenges, mounting apprehensions regarding the emission of greenhouse gases, particularly CO_2_, have garnered heightened scrutiny^1^. Therefore, extensive research efforts have been directed towards exploring alternative fuels, such as nuclear and renewable energy, to mitigate the world's reliance on fossil fuels. The emphasis is on identifying environmentally friendly energy options that rely on renewable sources^2–4^. While nuclear energy has been considered as an alternative, its resource availability is constrained, and effective waste disposal remains a prominent challenge^5^. As a result, renewable energy sources that offer minimal to zero waste discharge have garnered significant attention within the scientific community.

Microbial fuel cells (MFCs) are a distinct type of fuel cell that employ microorganisms as biocatalysts, converting organic matter into electricity by facilitating the transfer of electrons and protons. Unlike conventional fuel cells that rely on costly catalysts, MFCs utilize microorganisms within the anode chamber. The electrons generated by these microorganisms reach the anode electrode, either through a mediator or via direct transformation using nanowires or biofilms, before being transferred to the cathode surface through an external circuit. In the case of membrane-less single chamber MFCs (SCMFCs), protons permeate through the anolyte to reach the cathode electrode. At the cathode, oxygen molecules undergo reduction, resulting in the production of water^1^. However, despite the potential benefits of MFCs, several critical challenges impede their widespread application for real-world scenarios. These challenges include the efficiency of electricity generation, material costs associated with electrodes and separators, the need for simplicity and feasibility in design and operation, as well as maintenance costs and overall viability. In order to overcome these barriers, the adoption of MFCs has been considered a promising alternative to traditional fossil fuel-based energy production. Consequently, substantial efforts have been dedicated over the past two decades to address one or more of these challenges and enhance the practical applicability of MFCs. Different operating conditions^6–8^ and separate anode and cathode materials^9,10^, modified anode or cathode electrode^11^, flow process (batch and continuous)^2^, and microbial type^12–16^ have been studied to achieve high energy production in MFCs. Low-cost anode electrode material (e.g., stainless steel mesh^17^), the separator (e.g., Canvas cloth^18^), and biocathode (cathode electrode without metal catalyst^19^) were used to decrease the initial cost in MFCs. Different MFC structures^20,21^ were studied to design MFCs with simple structures and easy maintenance. Heretofore, two types of tubular MFCs have been utilized, up the flow (vertical)^22^ and horizontal^20^. Tubular MFCs can be used in real electricity production and wastewater treatment applications for easy maintenance and structural features (e.g., no dead end in continuous mode). Even in tubular types, the cost of producing electricity is still high and unjustifiable, which has limited their application so far.

Studying the effect of feed type (from simple to complex feeds) on the efficiency of the MFC may lead to identifying a proper design to eliminate this technology's long-lasting challenges and assist its commercialization. High voltage loss compared to commercial types is one of the most critical weaknesses of MFCs. Mathematical models can identify the main sources of voltage loss. Insufficient attention has been paid to structural changes in feed and simultaneous modeling of results in the literature.

This research study aims to comprehensively investigate the influence of substrate on the performance of membrane and mediator-less tubular single chamber microbial fuel cells (SCMFCs). The study utilizes two different types of carbon sources, namely synthetic sources (glucose and microcrystalline cellulose) and an industrial feed consisting of a slurry derived from the organic fraction of municipal solid waste (SOMSW), to assess the effect of substrate on MFC performance. The performance evaluation encompasses various aspects, including electrical parameters such as open circuit voltage (OCV) and power density in closed circuit mode, as well as different biological conditions such as pH levels and concentrations of volatile fatty acids (VFAs). The obtained results are then compared with existing literature to provide a comprehensive analysis. Additionally, a mathematical model is employed to calculate voltage losses within the system, thereby shedding light on the underlying mechanisms that impact the performance of the membrane and mediator-less tubular SCMFCs.

## Materials and methods

**Figure 1:**
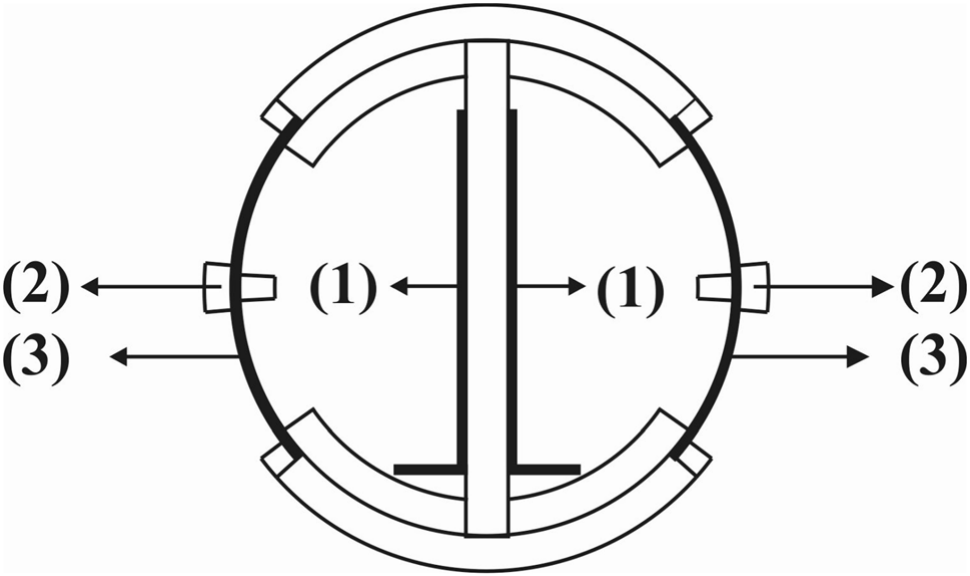
Cross section of anode chamber, 1: anode electrode, 2: protective porous plate, 3: cathode electrode.

Tubular membrane-less SCMFC was built of Plexiglas tube (inner diameter: 6 cm, outer diameter: 8 cm, and length: 17 cm) and a volume of 485 mL^23,24^. The anode electrode was fabricated by 24 × 6 cm stainless steel mesh. Graphite coating was carried out by spraying graphite paint on the surface of the stainless steel mesh^17^. Carbon clothes (E-TEK, USA, 64 cm^2^) were used as a cathode electrode, and its surface was modified to achieve about^25,26^ 0.5 mg-Pt/cm^2^, and 30 wt. % Nafion loading. The anode electrode is fixed on a plexiglass plate and located in the middle of the anode compartment. The cathode electrode was set on a protective porous plate and located on both sides of the anode electrode on the wall of the tube, as shown in Fig. 1. Anode and cathode electrodes were connected electrically using a copper wire.

In the summer, municipal solid waste (MSW) was collected from the Isfahan landfill site (32.71, 51.69, Isfahan, Iran). Metals and inorganic materials were separated from the MSW. Then, the organic fraction of MSW (OMSW) was dried. After that, OMSW was milled and sieved to a particle size between 833 to 177 μm.

The OMSW were mainly consisted of starch 56.0 ± 0.6%, lipid 6.0 ± 0.1%, protein 8.1 ± 0.4%, pectin 9.2 ± 0.8% and lignocelluloses 20.7 ± 0.5%. The solution of OMSW and microcrystalline cellulose (MCC) was stirred and heated at 80–90 °C for about one hour on a hot plate (MS300HS- Misung scientific) to give a suitable slurry, then cooled and used as the substrate. Anaerobic treatment rectors of the Isfahan wastewater treatment plant with total solid (TS) of 4.53 ± 0.13% and total volatile solid (TVS) of 2.18 ± 0.12% were utilized as the microorganism source. The SCMFCs were filled with a mixture of microorganisms and synthetic wastewater solution (carbon source 1 g/L, KCl 0.13 g/L, Na_2_HPO_4_ 4.1 g/L, NaH_2_PO_4_ 2.55 g/L, NH_4_Cl 0.31 g/L mineral solution 12 mL/L and vitamin solution 5 mL/L) with a ratio of 2:8. When the output voltage descended to the values about half of the last maximum voltage, anolyte replaced with a fresh substrate [glucose solution (1 g/L), MCC and SOMSW (2.5 g/L)]. The pH and temperature of feeds were set at 8.5 ± 0.1 and 23 ± 2 °C, respectively.

### Calculations and analysis

The pH of the anolyte was measured by a pH meter (SL 901, SANA, Iran) based on APHA 4500 standard method. The concentration of glucose was analyzed by high-performance liquid chromatography (HPLC) method with a refractive index (RI) detector (Jasco International Co., Tokyo, Japan) supplemented with ion-exchange column (Aminex HPX-87H, Bio-Rad, Richmond, CA, USA). Glucose was analyzed by ion-exchange column (Aminex HPX-87P, Bio-Rad, Richmond, CA, USA) with 0.6 mL/min deionized water as eluent at 80 °C.

The morphology of the biofilm formed on the anode electrode was investigated by a scanning electron microscope (Philips, XL30 SEM). The voltage was measured using a multimeter and recorded on a memory card by an analog-to-digital converter (ADC) board every 10 min. A series of external resistance from 100 kΩ to 10 Ω were used to produce a polarization curve. External resistance was changed when the output voltage reached its stable value (about 6 h in each external resistance).

The current density I_d_ (mA/m^2^) was calculated by Ohm law (I_d_ = V/(R_ex_*A_cat_)). *V* (mV), *R*_ext,_ (Ω), and A_Cat_ (m^2^) are the voltage, external resistance, and surface area of the cathode electrode, respectively. Power density, *P*_d_ (mW/m^2^), was calculated by Eq. (1).

1 $$P_{d} = VI_{d}$$

Coulombic efficiency (*CE*) was calculated using the following equation:

2 $$CE = \frac{{M\int_{0}^{{t_{b} }} {Idt} }}{{FbV_{An} \Delta C}}$$

where *I* (mA) is current, F is Faraday’s constant (96,500 C/mol), M is molecular weight (glucose 180.16 g/mol and oxygen 32 g/mol), b is the number of electrons exchanged per mol (glucose and oxygen 24 and 4 e^−^, respectively), *V*_An_ (L) is the volume of anolyte, ∆C/(g/L) is the overall change in concentration during the run duration, and *t*_b_ is run time (s).

Main anodic and cathodic reactions occurred in microbial fuel cell

$${\text{Anodic reaction}}\;({\text{glucose used as substrate}}):\quad {\text{C}}_{6} {\text{H}}_{12} {\text{O}}_{6} + 6{\text{H}}_{2} {\text{O}}\mathop{\longrightarrow}\limits^{{{\text{Microbes}}}}6{\text{CO}}_{2} + \, 24{\text{H}}^{ + } + 24{\text{e}}^{ - }$$

$${\text{Cathodic reaction:}}\quad {\text{O}}_{2} \, + \, 4{\text{H}}^{ + } + 4{\text{e}}^{ - } \mathop{\longrightarrow}\limits^{{\text{catalyst(Pt)}}}{\text{H}}_{2} {\text{O}}$$

Anodic reaction with polymers (large molecules (MCC and SOMSW)) differs from glucose as feed. Large molecules must break down (proteins and polysaccharides) into simple molecules (volatile fatty acids)^27^, and after that, simple substrates convert to electricity.

## Results and discussion

### Electrical performance

In the first cycle (start-up duration), three SCMFCs were operated at open circuit mode (OCM). At the start of the process, in SCMFC with glucose as substrate, three rising steps were observed for OCV. In the first step, OCV increased rapidly from 294 to 507 mV during 20 min (rising rate = 0.639 V/h), then the rate of rising decreased to 12.65 mV/h (from 507 to 642 mV), and in the last step, OCV increased to 695 mV during 16.5 h. The increasing trend of OCV continued until a limiting factor (the maximum ability of microorganisms, decrease in pH, or temperature) was accomplished. After that, the stationary phase was started, and the OCV value fluctuated between 536 and 665 mV. The same trend of OCV with temperature was observed in the stationary phase; OCV decreased (from 693 to 536 mV) with decreasing temperature (from 23 to 20.5 °C). Further, an increase in temperature to 23.5 °C increased the OCV to 664 mV. A maximum OCV value of 693 mV was attained in MFC with glucose (MFC-G) as substrate. The same trend was observed in three SCMFCs at OCM mode. Maximum OCV values of 550 and 520 mV were obtained for MFC-MCC and MFC-SOMSW systems.

**Figure 2:**
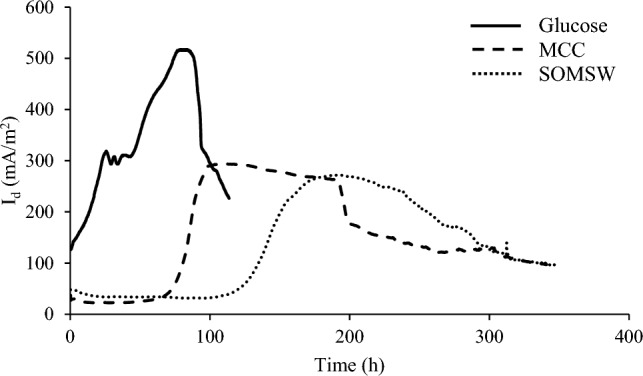
Current density produced in MFC at external resistance 100 Ω.

External resistance of 100 Ω was used as an electricity consumer in close circuit mode (CCM). Produced power density in MFCs is presented in Fig. 2. Accordingly, the rising phase in CCM had three regions when glucose was utilized as the carbon source. However, the increasing phase had different voltage trends at OCM and CCM. In the first region, current density raised from 131 to 317 mA/m^2^ during 25 h, then remained almost constant with a small variation between 317 and 293 mA/m^2^ during 48 h. Finally, current density achieved 517 mA/m^2^ (power density 171 mW/m^2^) during 15 h. Two mechanisms (which seem to be responsible for the three mentioned steps) have been defined for converting glucose to electricity by microorganisms, direct and indirect (glucose converted to middle products such as VFAs and middle product converted to electricity). A part of the substrate is directly converted to electricity by changing the feed source; consequently, current density increases (first step). Another part of the substrate was converted to middle products. Then electricity was produced by using a portion of the middle products as the substrate (the last step of the rising phase). It was found that the response of MFC to feed injection depends on feed complexity. Feed replacement caused a delay in output voltage reinforcement (as feedback to feed injection) in MFC-MCC and MFC-SOMSW cases. This delay may be attributed to the stage of converting large molecules (polymers) to middle products. Afterwards, these middle products are consumed by electrogenic bacteria and converted to electricity. The size of the polymers in SOMSW was higher than that of MCC. Higher molecular weight required more time for breaking down the polymers in SOMSW, and correspondingly, higher delay time was expected for MFC-SOMSW compared to MFC-MCC. The main results are shown in Table 1.

**Table 1:** Performance of MFC with different substrate.

	I_max_ (mA/m^2^)	P_max_ (mW/m^2^)	pH	R_int_ (Ω)	CE%
MFC-G	517.2	172.0	6.5 ± 0.1	130	18 ± 0.1
MFC-MCC	294.4	55.5	7.4 ± 0.1	280	
MFC-SOMSW	273.3	47.9	7.5 ± 0.1	332	8.4 ± 0.1

A decrease in pH of the anolyte from 8.5 to 6.5, 7.4, and 7.5 in MFCs with glucose, MCC, and SOMSW, respectively, were observed in the anode chamber in daily analysis. In the anode chamber, the produced carbon dioxide was dissolved in the anolyte and then converted to carbonic acid. pH variations may be attributed to VFAs and carbonic acid production in the anolyte. Some microorganisms consume glucose and produce VFAs, and other intermediates (Acidogenesis step), while others use VFAs in their metabolism to generate electricity or methane. At the start of the cycle (high substrate concentration), the production rate of VFAs was more than the consumption rate, resulting in the accumulation (positive net production) of VFAs. However, the concentration of the feeds decreased gradually, which led to negative values for the net production of VFAs. The removal and Coulombic efficiencies (CE) of glucose (calculated by Eq. 2) were 97 ± 1 and 18 ± 0.1%, respectively. Also, COD removal of 74% (after ten days) and CE of 8.43% in MFC with SOMSW were obtained. The results (COD removal, maximum power density, and CE) showed that treating wastes by MFC and direct electricity production could be considered an alternative process to typical methods.

**Figure 3:**
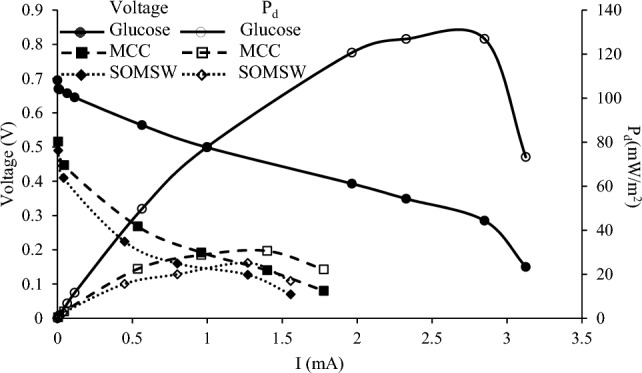
Polarization curve of SCMFC (voltage: left axis and power density: right axis).

Polarization curves produced by evolution OCV and the voltage across external resistances are shown in Fig. 3. According to Fig. 3, the polarization curve indicated three regions: activation, ohmic, and concentration loss^28^. The first region of the polarization curve is activation loss. In this region, a sharp decrease in voltage was observed. Activation energy corresponding to the cathode and anode reactions caused the activation loss. In the second part of the polarization curve, voltage decreased almost linearly by increasing the current. The last region in the polarization curve is concentration loss. The slope of the ohmic part in the polarization curve and optimum external resistance were used to calculate internal resistance, as depicted in Table 1. According to Table 1, internal resistances in MFC-G, MFC-MCC, and MFC-SOMSW were 130, 280, and 332 Ω, respectively, indicating that the internal resistance increased by increasing the substrate complexity. The highest internal resistance was observed utilizing MFC-SOMSW, which can be due to the complexity of SOMSW compared to the other substrates.

### Polarization curve modeling

As mentioned above, according to Eq. (3)^28–30^, the real voltage in FCs lost with three overvoltages relative to OCV as follows:

3 $${\text{V }} = {\text{ OCV}} - \eta_{act} - \eta_{ohmic} - \eta_{conc}$$

4 $${\text{V }} = {\text{ OCV}} - \left( {{\text{a}} \times \ln \left( {\frac{i}{{i_{0} }}} \right)} \right) \, - \, ({\text{R}}_{{{\text{ohmic}}}} \times S \times i) \, - \, \left( {{\text{C}} \times \ln \left( {\frac{{i_{l} }}{{i_{l} - i}}} \right)} \right)$$

In Eq. (3) *η*_*act*_, *η*_*ohmic*_ and *η*_*conc*_ referred to anode and cathode reaction activation (Tafel Eq.), ohmic (loss due to internal resistance like anolyte resistance, the distance between electrodes, etc.) and concentration voltage loss, defined by second, third, and fourth term in right-hand side of Eq. (4), respectively. In Eq. (4), S (m^2^), a (V), i_0_ (mA/m^2^), i_l_ (mA/m^2^), and R_ohmic_ (kΩ) are surface area, Tafel slope, exchange current density, maximum achievable current, and ohmic resistance, respectively. Equation (4) was assisted in modeling the polarization curve. Five fitting parameters, including a, i_0_, R_ohmic_, C, and i_l,_ were adjusted on the experimental data (produced in MFC with different substrates), resulting in relative errors lower than 10% and R^2^ higher than 0.98. The results of the modeling are shown in Table 2.

**Table 2:** Adjusted parameters.

Substrate	a (V)	i_0_ (mA/m^2^)	R_ohmic_ (kΩ)	C (V)	i_l_ (mA/m^2^)	ARE%	R^2^
Glucose	0.013	8.26	0.102	0.076	546.85	8.54	0.98
MCC	0.045	0.746	0.106	0.003	453.13	1.26	0.99
SOMSW	0.044	0.077	0.102	0.002	390.62	2.40	0.99

**Figure 4:**
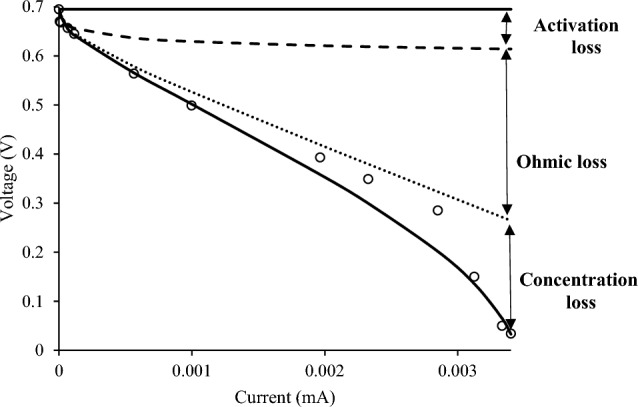
Calculated (solid line) and experimental (open circles) voltage.

The modeling results of MFC with glucose as substrate and the portion of each loss in voltage drop were presented in Fig. 4, and a good agreement between calculated and experimental data was observed. The percentage of activation loss in overall voltage drop in MFC-G, MFC-MCC, and MFC-SOMSW were 9.7, 56.6, and 78%, respectively. So, it can be concluded that the portion of activation loss increased with increasing substrate complexity. Thus, in real applications of MFC in industrial waste treatments, the source of the activation loss (electrochemical reaction kinetics) should be settled and kept under control. The exchange current density for either natural process should be high, which means higher exchange current density leads to lower voltage drop^29^. According to the modeling results, the estimated value of the exchange current density sequence was MFC-G > MFC-MCC > MFC-SOMSW. Thus, higher activation loss in MFC-SOMSW may be due to the lower exchange current density.

### Morphology of biofilm

Morphologies of the formed biofilm on the anode electrode were investigated using SEM graphs.

**Figure 5:**
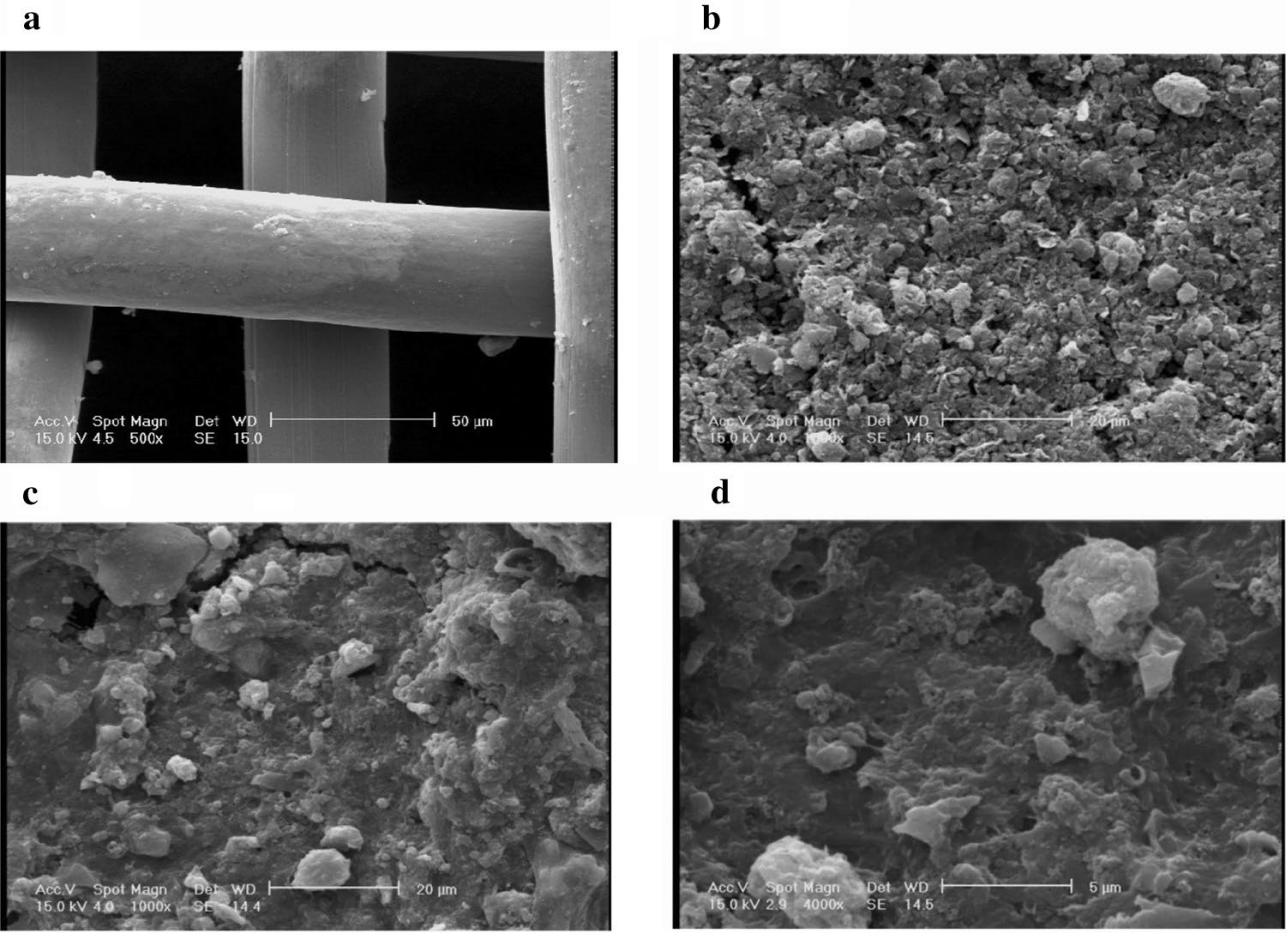
SEM graphs. (**a**) Stainless steel mesh, (**b**) graphite coated on stainless steel mesh, (**c**) anode electrode after biofilm formation with magnification 1000, (**d**) anode electrode after biofilm formation with magnification 4000.

SEM graphs of stainless steel mesh, graphite coated on stainless steel mesh, and anode electrode after biofilm formation are shown in Fig. 5. Accordingly, the anode electrode's surface area increased by forming the biofilm. Considering Fig. 5c,d, the appearance of the voids on the biofilm layer is obvious. It was found that these voids could be useful in two ways: (1) reinforcement of mass transfer from bulk anolyte to biofilm (by forming water channels that assist substrate transfer to underlayers of the biofilm), (2) transferring products, especially proton to anolyte.

### Comparison of the electrical results with the literature

Results of the current study and the literature (batch mode, similar substrate, and similar cell shape (tubular)) are compared in Table 3. Glucose has been used as a substrate in many studies. However, the main part of these studies was focused on materials or modification of electrodes^31^. According to Table 3, in the same order of anode chamber volume, the CE of MFC-G in the present study increased more than threefold, and the power density rose 50% compared to the average value of CE^32^ and power density^2^ in the literature. The membrane cost was about 30% of the initial cost of MFC, while the mixing technique (mixing with the magnetic stirrer or recirculation) or mediator in the batch mode MFCs involved a significant part of the operating cost. Despite the significant decrease in the total cost (initial cost and operating cost), the proper performance of the tubular MFC was mainly due to adequate surface contact between the anode electrode and the settled microorganisms.

**Table 3:** Comparison results of this research with literature (with the same flow method and anode volume scale).

Type	V_anode_ (mL)	Substrate	Separator	P_d_ (mW/m^2^)	CE%	References
SC*	550	Glucose	PEM**	180	6.2	^32^
SC	550	Glucose	PEM	123	4.75	^32^
SC	550	Glucose	PEM	92	2.89	^32^
DC***	615	Glucose	PEM	135	13	^2^
DC	346	Glucose	PEM	35	0.3	^33^
DC	300	Glucose	PEM	52	9.9	^34^
SC	485	Glucose	Membrane Less	171	18 ± 0.1	This work
DC	75	MCC	PEM	66	–	^35^
DC	75	MCC	PEM	3.5	–	^36^
SC	485	MCC	Membrane Less	55.5	–	This work
SC	250	SOMSW	PEM	31.6	3.6	^37^
SC	28	SOMSW	Membrane Less	123	24	^38^
DC	60	SOMSW	PEM	11.9	1.95	^39^
SC	485	SOMSW	Membrane Less	47.9	8.4 ± 0.1	This work

*Single chamber.

**Proton exchange membrane.

***Dual chamber.

Generally, electrical performance (current and power density) drop is a significant drawback in the scaling-up of MFCs. Besides, structural changes such as a broader gap between the electrodes (increased internal resistance) in large-scale manufacturing is, an important obstacle. Additionally, waste treatment must be proceeded on a large scale (more than 1000 m^3^/day or ton/day), requiring large-scale design of MFCs. Comparing the results of the current research and the presented small-scale tubular MFC in the literature revealed a successful scale-up. Moreover, the produced power density was approximately equal to the best-reported power density in tubular MFC with membrane^35^. Furthermore, in the case of MFC-SOMSW (with the largest MFC in literature), it produced power enhanced more than 50%^37^.

## Conclusion

The conducted research comprehensively investigated the electrical and biological performance of microbial fuel cells (MFCs) utilizing different substrates. Notably, MFC-G exhibited superior electrical performance compared to the other MFCs studied. The maximum power density achieved in MFC-G was 171 mW/m^2^, which was approximately three times higher than that of MFC-MCC and MFC-SOMSW. These electrical results indicate that MFCs can serve as viable alternatives to conventional waste treatment processes, even when operating with complex substrates such as natural polymers found in the slurry of the organic portion of municipal solid waste. Moreover, the modeling analysis revealed that increasing substrate complexity led to an escalation in activation loss. The modeling results indicated that the proportion of activation loss to the total loss in MFC-G, MFC-MCC, and MFC-SOMSW was 8.9%, 49%, and 52.2%, respectively. This implies that nearly half of the total loss in MFCs operating with complex feeds can be attributed to activation loss. These findings provide valuable insights into the performance and optimization of MFCs when utilizing different substrates. They contribute to the understanding of the factors influencing activation losses in MFCs, which can aid in the development of strategies to enhance overall efficiency and performance in the future.

## Data Availability

Data are available with the permission of [Milad Kadivarian]. The data supporting this study's findings are available from the corresponding author, [Milad Kadivarian], upon reasonable request.
